# Synthesis and structure of two novel *trans*-platinum complexes

**DOI:** 10.1107/S205252062300327X

**Published:** 2023-05-06

**Authors:** Doriana Vinci, Daniel Chateigner

**Affiliations:** a European XFEL GmbH, Holzkoppel 4, Schenefeld, Hamburg 22869, Germany; b CRISMAT, CNRS, ENSICAEN, Université de Caen Normandie, Caen, France; Georgetown University, USA

**Keywords:** platinum complexes, synthesis, anticancer drugs, X-ray diffraction, crystal structure

## Abstract

Here for the first time the synthesis and characterization of two new *trans*-platinum complexes, *trans*-[PtCl_2_{HN=C(OH)C_6_H_5_}_2_] (compound **1**) and *trans-*[PtCl_4_(NH_3_){HN=C(OH)^t^Bu}] (compound **2**) [with ^t^Bu = C(CH_3_)_3_] are described. The structures have been characterized using nuclear magnetic resonance spectroscopy and X-ray single-crystal diffraction. In compound **1** the platinum cation, at the inversion center, is in the expected square-planar coordination geometry. It is coordinated to two chloride anions, *trans* to each other, and two nitro­gen atoms from the benzamide ligands. The van der Waals interactions between the molecules produce extended two-dimensional layers that are linked into a three-dimensional structure through π⋯π intermolecular interactions. In compound **2** the platinum cation is octahedrally coordinated by four chloride anions and two nitro­gen atoms from the pivalamide and ammine ligands, in *trans* configuration. The molecular packing is governed by intermolecular hydrogen bonds and van der Waals interactions.

## Abstract 2

The synthesis and structural characterization (XRD and NMR) of two new *trans*-platinum complexes are presented.

## 1. Introduction

Platinum-based drugs have been used for chemotherapeutic treatment of cancer since 1965, when Rosenberg’s group discovered the cytotoxic activity of cisplatin Pt(NH_3_)_2_Cl_2_ (Rosenberg *et al.*, 1965 ▸). In 1970, cisplatin was approved for application in testicular and ovarian cancer by the US Food and Drug Administration and in several European countries (Wiltshaw, 1979 ▸). It is prescribed also for the treatment of a wide array of other tumors such us head-and-neck, esophagus, stomach, colon, bladder, cervix, pancreas, liver, kidney and prostate cancers. Although cisplatin has been used for more than 40 years, the severe side effects and the drug resistance of many cancer types have been the major limitations for its clinical application (Lippert, 1999 ▸; Giaccone, 2000 ▸; Oberoi *et al.*, 2013 ▸). Cisplatin resistance may be associated with reduced drug uptake, enhanced efflux, intracellular detoxification by gluta­thione, increased DNA repair, decreased mismatch repair, defective apoptosis, modulation of signaling pathways or the presence of quiescent non-cycling cells (Steward, 2007 ▸; Rabik & Dolan, 2007 ▸; Kuo *et al.*, 2007 ▸; Boulikas *et al.*, 2007 ▸; Heffeter *et al.*, 2008 ▸; Reedijk, 2011 ▸). In the last 45 years, although a great effort has been made for synthesizing platinum compounds with reduced side effects and propensity to induce drug resistance (Jakupec *et al.*, 2003 ▸), none of them has reached worldwide clinical application. Only five complexes (carboplatin, oxaliplatin, nedaplatin, heptaplatin, lobaplatin) have been registered for clinical treatment with regional approval (cisplatin, carboplatin and oxaliplatin are FDA-approved, nedaplatin in Japan and lobaplatin in China). According to early structure–property relationship studies, only platinum compounds with *cis*-configuration exhibit antitumor activity (Kelland, 2007 ▸). In recent decades, however, it has been observed also that many *trans*-platinum(II) complexes exhibit anticancer activity comparable with the *cis*-isomer and cisplatin (Farrell *et al.*, 1992 ▸; Montero *et al.*, 1999 ▸; Kasparkova *et al.*, 2003*a* ▸,*b* ▸). The promising biological activity of *trans*-platinum(II) complexes encouraged us to synthesize and characterize a novel *trans*-platinum(II) complex with a benzamide ligand, namely *trans*-{PtCl_2_[HN=C(OH)C_6_H_5_]_2_}. Derivatives of benzamide are known to possess cytotoxic activity (Vernhet *et al.*, 1997 ▸; Rauko *et al.*, 2001 ▸; Zhang *et al.*, 2022 ▸) and such a ligand is interesting also owing to the occurrence of a hydroxyl group which can serve as a hydrogen-bond donor or acceptor.

Although the platinum drugs currently used for cancer treatment consist of platinum cations with oxidation state +2, in recent years platinum(IV) species have also been investigated. The interest in Pt^IV^ complexes arises from their greater inertness to ligands substitution compared with Pt^II^ counterparts, a feature that allows chemical modification of the ligands without breaking the metal–ligand bond. The slow exchange rate of ligands coordinated to Pt^IV^ plays an increasingly important role for the development of new nanotechnology for delivering platinum drugs to cancer cells (Dhar *et al.*, 2011 ▸; Min *et al.*, 2010 ▸). The presence of two extra coordination sites can also be used in combination with other drugs, or for modifying biological targets other than DNA in the cell. In addition, platinum(IV) complexes are stable in the oxidizing extracellular environment and they can easily reach the platinum(II) oxidation state inside the cell (Wong & Giandomenico, 1999 ▸). The increasing interest for Pt^IV^ species prompted us to extend our investigation to a platinum(IV) complex, *trans*-[PtCl_4_(NH_3_){HN=C(OH)^t^Bu}]. The bulky substituent (tertiary butyl group) in the amide ligand could potentiate the cellular uptake of the complex *via* passive diffusion through the cell membrane, because of the greater affinity for lipophilic environments, while the hydroxyl group would preserve the water solubility.

## 2. Experimental

### 2.1. *trans*-[PtCl_2_{HN=C(OH)C_6_H_5_}_2_] synthesis

**Figure 1:**
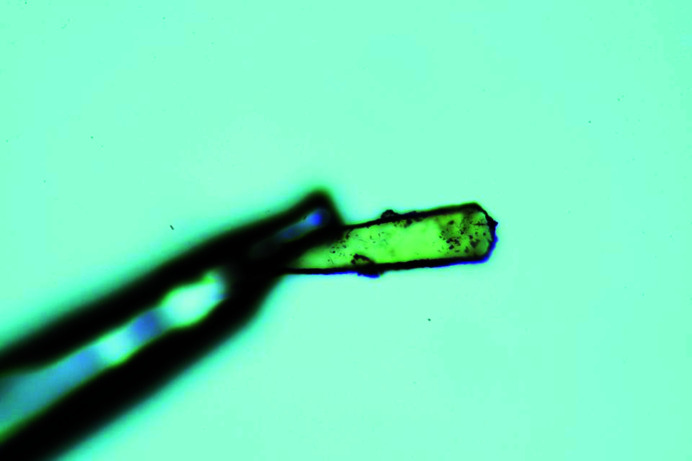
Crystal of *trans*-[Pt^II^Cl_2_{HN=C(OH)C_6_H_5_}_2_] complex selected for X-ray diffraction measurement.

Compound **1** was prepared by protonation of the K_2_{*trans*-[Pt^II^Cl_2_(H_2_NC(=O)C_6_H_5_)_2_]} salt: the reactant (0.6 g, 1 mmol) was dissolved in ice-cold water and treated with an excess of hydro­chloric acid (10 ml, 6 M). The yellow precipitate separated from the solution was collected by filtration of the mother liquor, washed with ice cold water and dried in a stream of dry air. Compound **1** was isolated, crystallized in chloro­form giving yellow lamellar crystals (Fig. 1 ▸) and then characterized using NMR spectroscopy and X-ray diffraction.

### 2.2. *trans*-[PtCl_4_(NH_3_){HN=C(OH)^t^Bu}] synthesis

The *trans*-[Pt^II^Cl_2_(NH_3_)(NC^t^Bu)] precursor [0.1780 g, 0.49 mmol, *M*_r_ (molecular weight) = 366 g mol^−1^] was suspended in chloro­form (30 ml) and Cl_2_ (2 ml), and stirred at 293 K for 30 min. The resulting solution was taken to dryness under reduced pressure, giving a yellow precipitate of *trans*-[Pt^IV^Cl_4_(NH_3_)(NC^t^Bu)]. The obtained complex was treated with KOH, then neutralized with HCl. The complex was isolated, crystallized in a mixture of chloro­form/pentane, and characterized by NMR spectroscopy followed by X-ray diffraction.

### 2.3. X-ray single crystal determination

Reflections were collected on a Bruker AXS X8 APEX CCD diffractometer equipped with a four-circle Kappa goniometer and a 4K CCD detector (Mo *Kα* radiation). Data reduction and unit-cell refinement were carried out with the *SAINT* package (Bruker, 2003 ▸). The reflections were indexed, integrated and corrected for Lorentz, polarization and absorption effects with the program *SADABS* (Sheldrick, 2010 ▸). All calculations and molecular graphics were carried out using *SIR92* (Altomare *et al.*, 1993 ▸), *PARST97* (Nardelli, 1995 ▸), *WinGX* (Farrugia, 1999 ▸), *CRYSTALS* (Carruthers *et al.*, 2003 ▸), *MERCURY* (Macrae *et al.*, 2020 ▸) and *ORTEP-3 for Windows* packages (Farrugia, 2012 ▸). Details of the experiment and crystal data are given in Table 1 ▸. Selected bond lengths and angles are listed in Table 2 ▸.

The unit-cell parameters were calculated from all reflections. Anisotropic displacement parameters (ADPs) for hydrogen and all non-hydrogen atoms were refined isotropically and anisotropically, respectively. The crystal structure was solved using direct methods in space groups 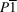 and *P*2_1_ for compounds **1** and **2**, respectively, and the models were refined using full-matrix least-squares.

#### 2.3.1. Compound **1**

**Figure 2:**
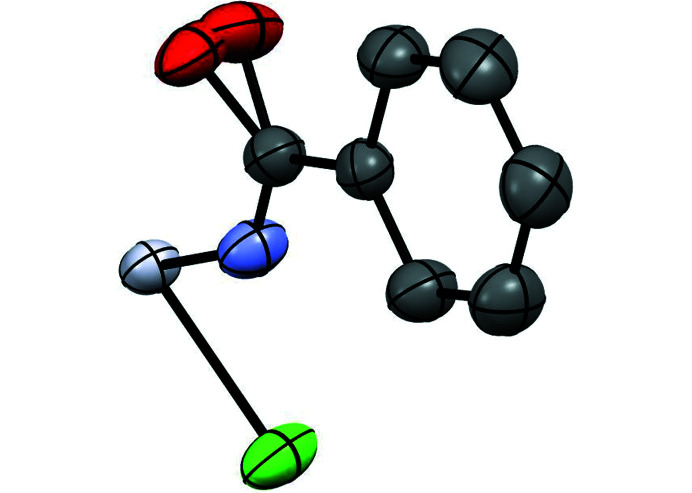
Oxygen splitting in compound **1**. Color coding for atoms: red: oxygen; dark gray: carbon; violet: nitro­gen; light grey: platinum; green: chloride.

The difference Fourier synthesis shows one maximum at the midpoint of two oxygen positions with the refinement resulting in grossly anisotropic displacement parameters corresponding to a ‘cigar-shaped’ ellipsoid. The disorder is refined using the so-called split-atom model strategy. The two partial atoms are refined independently, even with the sum of their site occupancies constrained to unity (Fig. 2 ▸). The hydrogen atoms were located by Fourier difference except for the hydrogen atoms located on oxygen sites which were placed at calculated positions. All hydrogen atoms were refined isotropically.

#### 2.3.2. Compound **2**

**Figure 3:**
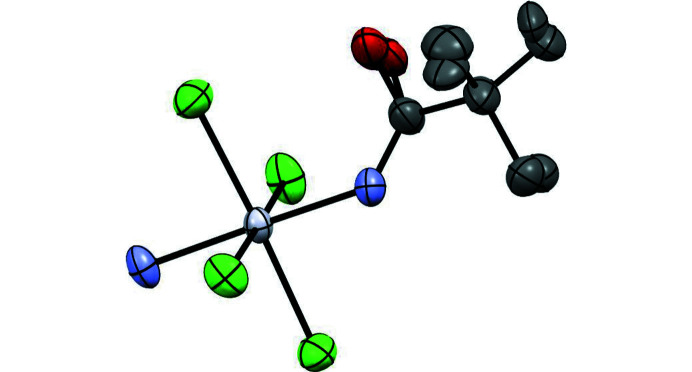
Oxygen and methyl splitting in compound **2** *a*. Color coding for atoms: red: oxygen; dark gray: carbon; violet: nitro­gen; light grey: platinum; green: chloride.

**Figure 4:**
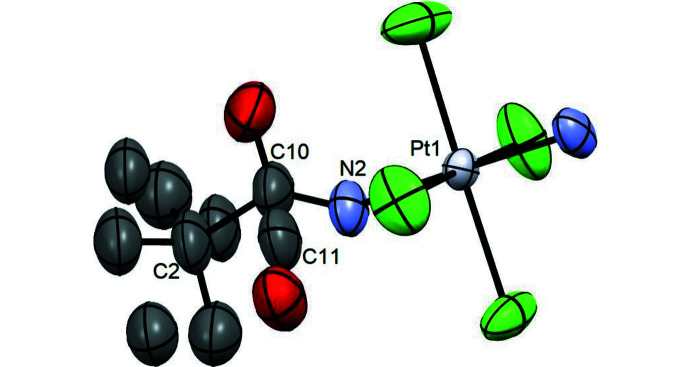
Amide ligand splitting in compound **2** *b*. Color coding for atoms: red: oxygen; dark gray: carbon; violet: nitro­gen; light grey: platinum; green: chloride.

The asymmetric unit includes two disordered complexes: the disorder of the first complex (*a*) involves the methyl in the tertiary butyl group [–C(CH_3_)_3_] and the oxygen atom in the amide moiety (NCO). The split-atom model has been used to model the disorder (Fig. 3 ▸). Since the hydrogen atoms bound to the split oxygen atoms have not been found by Fourier difference, they were added manually and refined isotropically. In the second complex (*b*) in the asymmetric unit of compound **2** shows an electron density symmetrically distributed around a a local plane through the N2 and C2 atoms of the pivalo­amide group. The disorder was modeled by splitting the C1 carbon atom and the tertiary butyl group (Fig. 4 ▸).

## 3. Results and discussion

### 3.1. NMR spectroscopy

#### 3.1.1. Compound **1**: platinum(II)

**Figure 5:**
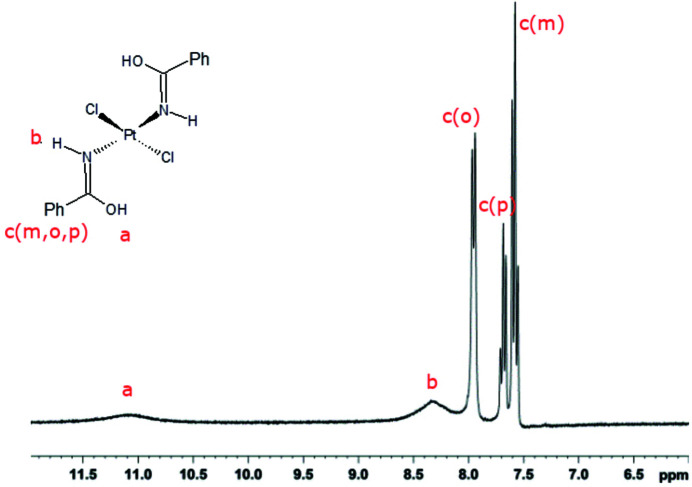
^1^H-NMR spectrum of compound **1**. Chemical shift (ppm): a δ[OH] ∼11.08, b δ[NH] ∼8.31, c δ[Ph(*o*)] ∼7.96, δ[Ph(*p*)] ∼7.68, δ[Ph(*m*)] ∼7.58. (*m* = *meta*, *o* = *ortho*, *p* = *para*).

The H^1^-NMR spectrum was recorded on a Bruker Avance DPX 300 MHz WB instrument at 295 K in acetone-d_6_. ^1^H chemical shifts were referenced to TMS by using the residual protic peak of acetone-d_6_ as internal reference. The ^1^H-NMR spectrum (Fig. 5 ▸) shows two broad signals at ∼11.08 ppm and ∼8.31 ppm assigned to the OH and NH protons in the amide ligand, respectively, and three aromatic proton contributions at 7.96 ppm, 7.68 ppm and 7.58 ppm from the *ortho*, *para* and *meta* protons, respectively.

#### 3.1.2. Compound **2**: platinum(IV)

**Figure 6:**
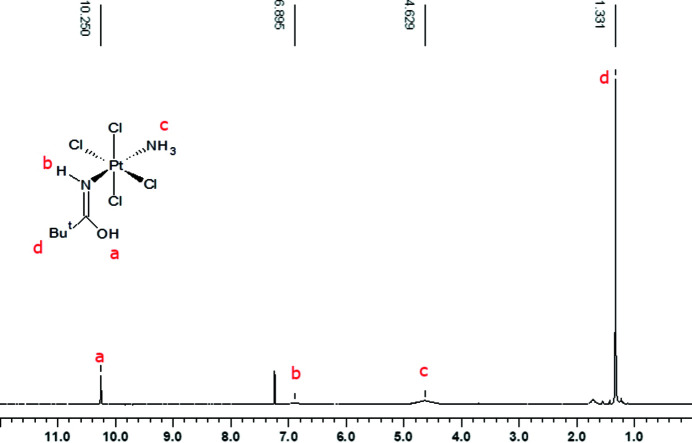
^1^H-NMR spectrum of compound **2**. Chemical shift (ppm): a δ[OH] ∼10.25, b δ[NH] ∼6.9, c δ[NH_3_] ∼4.63 and d δ[*tert*-butyl] ∼1.33.

^1^H-NMR spectrum was collected at 295 K in CDCl_3_. ^1^H chemical shifts were referenced to TMS by using the residual protic peak of CDCl_3_ as internal reference. The ^1^H-NMR spectrum (Fig. 6 ▸) contains a sharp signal at ∼1.33 δ and a broad signal at ∼4.63 δ assigned to the *tert*-butyl group and NH_3_ protons, respectively, in good agreement with a previous work on a *trans*-Pt^III^ complex with similar ligands {δ[*tert*-butyl] ∼1.20, δ[NH_3_] ∼5.00 in Vinci & Chateigner (2022 ▸)}. Moreover, two single proton resonances at ∼6.90 δ and 10.25 δ can be assigned to the NH and OH groups in the amide moiety. It is worth noting that the shape of the hydroxyl proton peak depends upon the nature of *R* [–C(CH_3_)_3_ in compound **2** or —C_6_H_5_ in compound **1**]: the signal is sharp for the ^t^Bu derivative and broader for the phenyl group. This feature may be explained by the chemical exchange process involving the hydroxyl proton and water impurities. The exchange rate is expected to increase with the acidity of the hydroxyl proton.

### 3.2. X-ray crystal structures

#### 3.2.1. Compound **1**: platinum(II)

**Figure 7:**
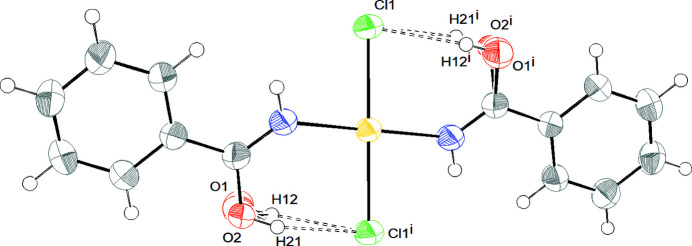
*ORTEP* drawing of compound **1** with intramolecular hydrogen bond interactions: [O1⋯Cl1 2.978 Å, H12⋯Cl1 2.184 Å, O1—H12⋯Cl1 163.56°] and [O2⋯Cl1 2.956 Å, H21⋯Cl1 2.165 Å, O2—H21⋯Cl1 162.04°]. The ellipsoids enclose 30% probability. Color coding for atoms: white: hydrogen; green: chlorine; blue: nitro­gen; gray: carbon; red: oxygen. Symmetry code (i): −*x* + 1, −*y* + 2, −*z*.

The asymmetric unit comprises half a molecule of the *trans*-[PtCl_2_{HN=C(OH)C_6_H_5_}_2_] complex. The structure is composed of one platinum cation, coordinated by two chloride anions and two benzamide ligands. The central platinum atom lies on the crystallographic inversion center in a slightly distorted square planar coordination geometry (Fig. 7 ▸). This distortion is due to the differences in the metal–ligand bond lengths as reported for analogous complexes (Fabijańska *et al.*, 2015 ▸). The N1, C1, C2 and O1 ligand atoms are coplanar as expected owing to the *sp* ^2^ hybridization of the N1 and C1 atoms linked with a double bond. The Pt—N bond distance length, 2.0072 (18) Å, agrees with previously reported values for *trans*-complexes with similar ligands [Pt—N from the literature: 2.01 (2)–2.067 (4) Å; Fabijańska *et al.*, 2015 ▸; Grabner & Bukovec, 2015 ▸; Cini *et al.*, 1999 ▸]. The lengths of the C1—N1, C1—O1 and C1—O2 bonds [1.274 (3), 1.325 (6) and 1.327 (7) Å, respectively] are shorter than those found for the corresponding single bonds, but larger than double bonds [from literature: C(*sp* ^3^)—N(*sp* ^3^) = 1.469 (14) Å, C(*sp* ^2^)=N(*sp* ^2^) = 1.279 (8) Å, C(*sp* ^3^)—OH = 1.426 (11) Å, C(*sp* ^2^)=O = 1.210 (8) Å; Allen *et al.*, 1987 ▸]. This means that a double bond is delocalized over the N—C—O moiety. The same behavior has already been reported for *N*-coordinated amidato (Erxleben *et al.*, 1994 ▸) and imino­ether ligands (Casas *et al.*, 1991 ▸). The length of the platinum–chloride bond, 2.3084 (6) Å, is nearly the same as found in complexes with the same *trans* influence. For example, in *trans*-Pt(3-af)_2_Cl_2_ [where 3-af = 3-amino­flavone (3-amino-2-phenylchromen-4-one, C_15_H_11_NO_2_) the Pt—Cl bond is 2.298 (1) Å; in *trans*-[PtCl_2_(dmso)*L*] [where *L* = 3-(pyridin-2-yl­methyl)­oxazolidin-2-one], the Pt—Cl bonds are 2.2965 (9) and 2.3025 (8) Å, whereas in *trans*-[PtCl_2_{HN=C(OH)C(CH_3_)_3_}_2_] an average value of 2.299 (3) Å is observed (Fabijańska *et al.*, 2015 ▸; Van Beusichem & Farrell, 1992 ▸; Cini *et al.*, 1999 ▸). The Pt1—N1—C1 angle is ∼137 (2)°, which is well above the expected values (120°), in agreement with the values observed for *trans*-[PtCl_2_{HN=C(OH)C(CH_3_)_3_}_2_] and *trans*-[PtCl_2_{HN=C(OMe)^t^Bu}_2_] complexes (Cini *et al.*, 1999 ▸). The benzamide plane and the platinum coordination plane, PtN_2_Cl_2_, make a dihedral angle of 20.86°. This orientation optimizes the intramolecular hydrogen bond interaction within the molecule.

The hydroxyl group points toward the chloride ligand resulting in an intramolecular hydrogen bond that stabilizes the complex (Fig. 7 ▸).

**Figure 8:**
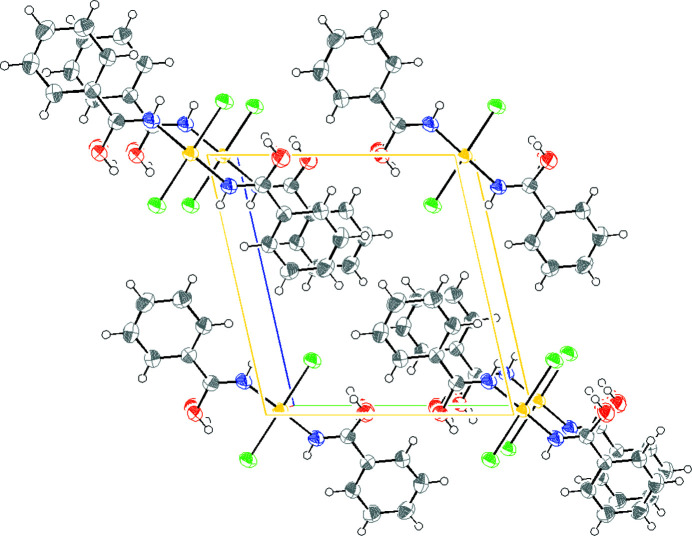
Molecular packing of compound **1**, view normal to the (100). Color coding for atoms: white: hydrogen; green: chlorine; blue: nitro­gen; gray: carbon; red: oxygen.

The molecular crystal packing is mainly governed by π⋯π stacking interactions and van der Waals intermolecular forces, involving the benzene ring of adjacent molecules with an intermolecular distance of ∼3.62 Å (Sinnokrot & Sherrill, 2006 ▸). The van der Waals intermolecular interactions involve hydrogen of the benzene ring and oxygen atom of the hydroxyl group [C7⋯O1*A* 3.280 (3) Å, H31⋯O2 2.613  (1) Å, C3—H31⋯O2 146.03 (8)°], resulting in the crystal packing shown in Fig. 8 ▸.

#### 3.2.2. Compound **2**: platinum(IV)

**Figure 9:**
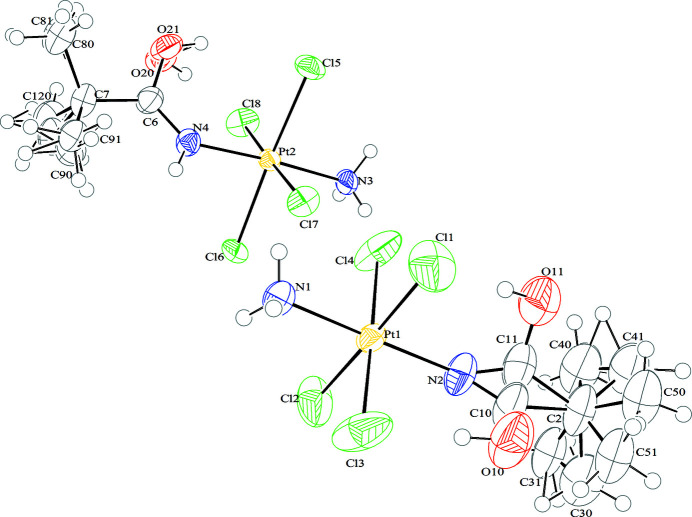
*ORTEP* diagram of two enantiomers of compound **2**. The ellipsoids enclose 30% probability. Color coding for atoms: white: hydrogen; green: chlorine; blue: nitro­gen; gray: carbon; red: oxygen.

**Figure 10:**
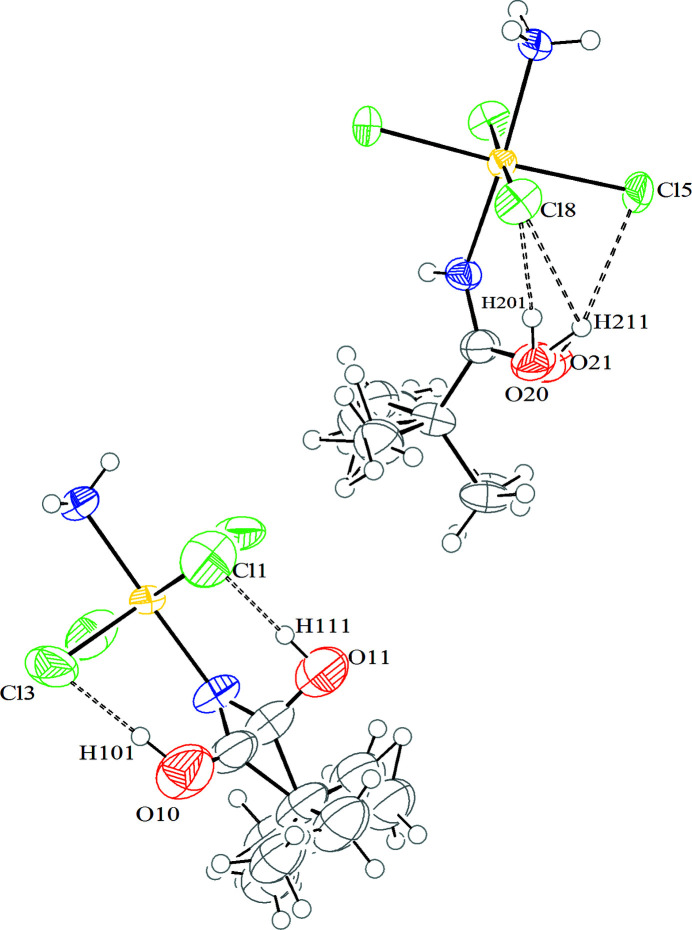
*ORTEP* drawing of two molecules of compound **2** linked by intramolecular hydrogen bonds (Table 3 ▸). The ellipsoids enclose 30% probability. Color coding for atoms: white: hydrogen; green: chlorine; blue: nitro­gen; gray: carbon; red: oxygen.

The crystal structure features two enantiomers in the cell [Flack parameter = 0.47 (1)]. The Pt^IV^ atom has an octahedral coordination geometry with four chloride ligands and two nitro­gen atoms (with hybridization *sp^2^* and *sp^3^* for amide and ammine ligands, respectively) in *trans* configuration (Fig. 9 ▸). The bond distances between platinum and ligands (Pt—N and Pt—Cl distances in Table 2 ▸) are in good agreement with values found previously for compound **1** and in the literature for platinum(III) and platinum(II) complexes (Vinci & Chateigner, 2022 ▸; Fabijańska *et al.*, 2015 ▸; Grabner & Bukovec, 2015 ▸; Cini *et al.*, 1999 ▸; Van Beusichem & Farrell, 1992 ▸). In the amide ligand, the C—N and C—O distances average 1.20 (7) Å and 1.13 (1) Å, respectively, due to the double bond delocalization over the N—C—O moiety as observed also for compound **1**. The C6—N4—Pt2, Pt1—N2—C11 and Pt1—N2—C10 angles are 132.7 (5)°, 140.2 (5)° and 141.3 (4)°, respectively, similar to the angle found for compound **1**. The larger angle is probably due to intramolecular hydrogen bonds involving the hydroxyl hydrogen and the chloride ligand (Fig. 10 ▸). The bond angles within the coordination sphere deviate significantly from the ideal value of 90°. For instance, in compound **2** with the atom labeled Pt2 (Fig. 9 ▸), two angles are particularly large [93.67 (19)° and 93.60 (17)°] for N4—Pt2—Cl5 and N4—Pt2—Cl8 angles, respectively) and two are particularly small [87.57 (16)° and 86.60 (19)°] for N3—Pt2—Cl6 and N3—Pt2—Cl8 angles, respectively). This feature is due to the hydrogen bond interactions between the hydroxyl group and the chloride ligands. The same behavior has been observed in the second enantiomer in the unit cell.

**Figure 11:**
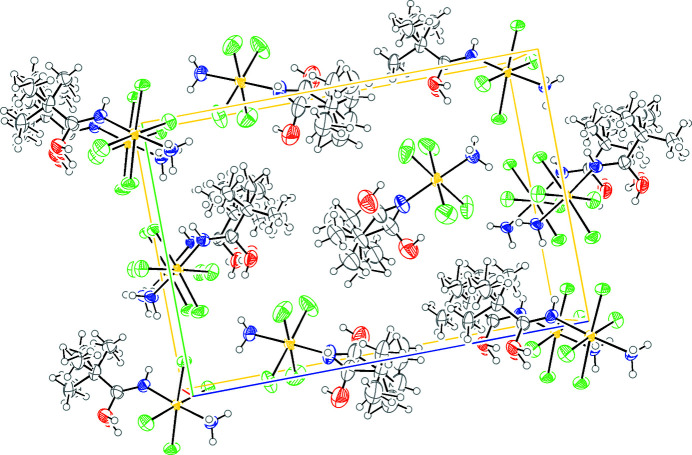
*ORTEP* drawing of packing of compound **2** governed by intermolecular hydrogen bonds and van der Waals interactions. The ellipsoids enclose 30% probability. Atom color coding: white for hydrogen, green for chlorine, blue for nitro­gen, gray for carbon and red for oxygen.

The molecular packaging (Fig. 11 ▸) is governed by hydrogen bonds and van der Waals interactions (Table 3 ▸). The intermolecular hydrogen bonds are observed between amide and chloride ligands. The intermolecular van der Waals interactions occur between the *tert*-butyl groups and the chloride ligands.

## 4. Conclusions

For compound **1** we observed that the protonation of K_2_{*trans*-[PtCl_2_(H_2_NC(=O)C_6_H_5_)_2_]} salt in ice-cold water, treated with an excess of hydro­chloric acid, gives the new *trans*-[PtCl_2_{HN=C(OH)C_6_H_5_}_2_] complex stable at room temperature. Spectroscopic studies indicate that the benzamide ligand is present in compound **1**. The X-ray structural determination confirmed that the central platinum(II) atom is four-coordinated *via* two nitro­gen atoms of the benzamide ligands and two chloride anions. The dihedral angle between PtCl_2_N_2_ and the benzene ring plane is 21 (1)°. The crystal lattice framework is governed by π⋯π and van der Waals intermolecular interactions.

Compound **2** was prepared by neutralization with HCl of a *trans*-[PtCl_4_(NH_3_)(NC^t^Bu)] solution in KOH. As expected, the X-ray structure contains a platinum(IV) atom six-coordinated by an ammine, four chloride and a pivalamide ligands with *trans* configuration. NMR spectroscopy confirmed the presence of the pivalamide ligand and the octahedral geometry.

A common feature between these two structures is the occurrence of intramolecular hydrogen bonds between the hydroxyl group and the chloride ligand. The next step in this work is the evaluation of the cytotoxic effect of these new platinum compounds against human and murine cancer cell lines, as well as the toxicity towards healthy cells and these effects will be compared with those of other cisplatin compounds.

## Supplementary Material

Crystal structure: contains datablock(s) global, 1, 2. DOI: 10.1107/S205252062300327X/yv5009sup1.cif

Structure factors: contains datablock(s) 1. DOI: 10.1107/S205252062300327X/yv50091sup2.hkl

Structure factors: contains datablock(s) 2. DOI: 10.1107/S205252062300327X/yv50092sup3.hkl

CCDC references: 2255221, 2255222

## Figures and Tables

**Table 1:** Experimental details

	Compound **1**	Compound **2**
Crystal data		
Chemical formula	*trans*-[PtCl_2_{HN=C(OH)C_6_H_5_}_2_]	*trans-*[PtCl_4_(NH_3_){HN=C(OH)^t^Bu}]
*M*_r_	508.27	455.08
Crystal system, space group	Triclinic, *P* 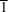	Monoclinic, *P*2_1_
Temperature (K)	293	293
*a*, *b*, *c* (Å)	4.1881 (1), 9.4591 (2), 10.1852 (2)	6.0583 (2), 12.1949 (4), 17.7364 (5)
α, β, γ (°)	102.793 (1), 91.304 (1), 100.914 (1)	90, 97.302 (2), 90
*V* (Å^3^)	385.48 (2)	1299.74 (7)
*Z*	1	4
Radiation type	Mo *K*α	Mo *K*α
No. of reflections for cell measurement	7402	9512
μ (mm^−1^)	9.45	11.59
Density (g cm^−3^)	2.181	2.325
Crystal size (mm)	0.36 × 0.06 × 0.02	Not measured

Data collection		
Diffractometer	Bruker AXS X8 APEX CCD	Bruker AXS X8 APEX CCD
Absorption correction	Multi-scan (*SADABS*)	Multi-scan (*SADABS*)
*T*_min_, *T*_max_	0.03, 0.05	1.00, 1.00
θ range (°) for data collection	2.06–39.88	3.47–36.34
No. of measured, independent and observed [*I* > 2.0σ(*I*)] reflections	17097, 4472, 3466	32159, 6519, 5194
*R*_int_	0.032	0.049
(sin θ/λ)_max_ (Å^−1^)	0.902	0.834

Refinement		
*R*[*F* ^2^ > 2σ(*F* ^2^)], *wR*(*F* ^2^), *S*	0.028, 0.021, 1.09	0.029, 0.031, 1.03
No. of reflections	3134	5204
No. of parameters	107	319
No. of restraints	16	209
H-atom treatment	H-atom parameters constrained	H atoms treated by a mixture of independent and constrained refinement
Δρ_max_, Δρ_min_ (e Å^−3^)	0.61, −0.52	2.07, −1.13

Computer programs: *COLLECT* (Nonius, 2001 ▸), *DENZO*/*SCALEPACK* (Otwinowski & Minor, 1997 ▸), *CrysAlis* (Oxford Diffraction, 2002 ▸), *SUPERFLIP* (Palatinus & Chapuis, 2007 ▸), *CRYSTALS* (Betteridge *et al.*, 2003 ▸), *CAMERON* (Watkin *et al.*, 1996 ▸).

**Table 2:** Selected bond lengths and angles (Å, °) for compound **1** and **2**

Compound **1**			
Cl1—Pt1	2.3084 (6)	N1—C1	1.274 (3)
O1—C1	1.325 (6)	N1—Pt1	2.0072 (18)
O2—C1	1.327 (7)		
O2—C1—C2	112.5 (6)	N1—Pt1—Cl1	84.05 (6)
O1—C1—C2	114.8 (4)	N1^i^—Pt1—Cl1^i^	84.05 (6)
O2—C1—N1	120.9 (6)	N1—Pt1—Cl1^i^	95.95 (6)
O1—C1—N1	119.3 (6)	N1^i^—Pt1—Cl1	95.95 (6)
N1—C1—C2	124.5 (2)	C1—N1—Pt1	136.41 (17)

Compound **2**			
Cl5—Pt2	2.3204 (16)	Cl1—Pt1	2.307 (3)
Cl6—Pt2	2.3070 (15)	Cl2—Pt1	2.291 (3)
Cl7—Pt2	2.3041 (17)	Cl3—Pt1	2.295 (3)
Cl8—Pt2	2.3228 (18)	Cl4—Pt1	2.318 (2)
N3—Pt2	2.054 (5)	N1—Pt1	2.038 (7)
N4—Pt2	2.022 (5)	N2—Pt1	2.035 (6)
C6—N4	1.279 (7)	C10—O10	1.373 (8)
C6—O21	1.322 (9)	C11—O11	1.364 (10)
C6—O20	1.323 (9)	C10—N2	1.166 (7)
		C11—N2	1.152 (8)
N3—Pt2—N4	176.5 (3)	N1—Pt1—Cl1	87.9 (3)
N3—Pt2—Cl8	86.60 (19)	N1—Pt1—Cl2	91.4 (3)
N4—Pt2—Cl8	93.60 (17)	N1—Pt1—Cl3	88.0 (3)
N3—Pt2—Cl6	87.57 (16)	N1—Pt1—Cl4	91.0 (3)
N4—Pt2—Cl6	88.94 (18)	Cl4—Pt1—Cl3	178.93 (14)
Cl8—Pt2—Cl6	90.61 (7)	Cl1—Pt1—Cl3	91.81 (16)
N3—Pt2—Cl7	90.67 (19)	Cl3—Pt1—Cl2	89.53 (17)
N4—Pt2—Cl7	89.14 (17)	Cl4—Pt1—Cl2	89.97 (15)
Cl8—Pt2—Cl7	177.27 (8)	Cl1—Pt1—N2	92.3 (2)
Cl6—Pt2—Cl7	89.48 (7)	Cl2—Pt1—N2	88.4 (2)
N3—Pt2—Cl5	89.81 (17)	Cl3—Pt1—N2	93.3 (2)
N4—Pt2—Cl5	93.67 (18)	Cl4—Pt1—N2	87.6 (2)
Cl8—Pt2—Cl5	90.13 (8)	N1—Pt1—N2	178.6 (3)
Cl6—Pt2—Cl5	177.24 (7)	Cl1—Pt1—Cl2	178.47 (16)
Cl7—Pt2—Cl5	89.65 (8)	Cl4—Pt1—Cl1	88.68 (14)
N4—C6—O21	121.5 (6)	O10—C10—N2	114.09 (10)
N4—C6—O20	121.1 (6)	O11—C11—N2	114.05 (10)
		N2—C10—C2	134.04 (10)
		N2—C11—C2	134.04 (10)
		Pt1—N2—C10	141.3 (4)
		Pt1—N2—C11	140.2 (5)

Symmetry code: (i): −*x* + 1, −*y* + 2, −*z*.

**Table 3:** Intra and intermolecular hydrogen bonds, and intermolecular van der Waals interactions (Å, °) for compound **2**

*D*—H⋯*A*	H⋯*A*	*D*⋯*A*	*D*—H⋯*A*
Intramolecular hydrogen bonds			
O10—H101⋯Cl3	1.93	2.90 (2)	179
O11—H111⋯Cl1	1.96	2.94 (5)	179
O10—H101⋯Cl3	2.01	2.96 (7)	159
O21—H211⋯Cl5	2.62	3.25 (3)	131
			
Intermolecular hydrogen bonds			
N1—H12⋯Cl8i	2.74	3.60 (3)	166
N1—H13⋯Cl7^ii^	2.66	3.48 (1)	154
N3—H31⋯Cl7^iii^	2.73	3.57 (6)	158
N3—H32⋯Cl6^iv^	2.49	3.32 (2)	156
N3—H33⋯Cl4^iii^	2.54	3.25 (4)	139
			
Intermolecular van der Waals interactions			
C40—H401⋯Cl1^iii^	2.68	3.46 (5)	139
C80—H803⋯Cl8^ii^	2.68	3.361 (2)	139

Symmetry codes: (i) −*x*, *y* + ½, −*z*; (ii) *x* − 1, *y*, *z*; (iii) *x* + 1, *y*, *z*; (iv) −*x* + 1, *y* − ½, −*z*.

## References

[bb1] Allen, F. H., Kennard, O., Watson, D. G., Brammer, L. & Orpen, A. G. & Taylor, R. (1987). *J. Chem. Soc. Perkin Trans. 2*, pp. S1–S19.

[bb2] Altomare, A., Cascarano, G., Giacovazzo, C., Guagliardi, A., Camalli, M., Burla, M. C. & Polidori, G. (1993). *Acta Cryst.* A**49**, *c*55.

[bb55] Betteridge, P. W., Carruthers, J. R., Cooper, R. I., Prout, K. & Watkin, D. J. (2003). *J. Appl. Cryst.* **36**, 1487–1487.

[bb3] Boulikas, T., Pantos, A., Bellis, E. & Christofis, P. (2007). *Cancer Ther.* **5**, 537–583.

[bb4] Bruker (2003). *SAINT*. Bruker AXS Inc., Madison, Wisconsin, USA.

[bb6] Casas, J. M., Chisholm, M. H., Sicilia, M. V. & Streib, W. E. (1991). *Polyhedron*, **10**, 1573–1578.

[bb7] Cini, R., Cavaglioni, A., Intini, F. P., Fanizzi, F. P., Pacifico, C. & Natile, G. (1999). *Polyhedron*, **18**, 1863–1868.

[bb8] Dhar, S., Kolishetti, N., Lippard, S. J. & Farokhzad, O. C. (2011). *Proc. Natl Acad. Sci. USA*, **108**, 1850–1855. 10.1073/pnas.1011379108 PMC3033286 21233423

[bb9] Erxleben, A., Mutikainen, I. & Lippert, R. (1994). *J. Chem. Soc. Dalton Trans.* pp. 3667–3675

[bb23] Fabijańska, M., Studzian, K., Szmigiero, L., Rybarczyk-Pirek, A. J., Pfitzner, A., Cebula-Obrzut, B., Smolewski, P., Zyner, E. & Ochocki, J. (2015). *Dalton Trans.* **44**, 938–947. 10.1039/c4dt01501k 25110914

[bb10] Farrell, N., Kelland, L. R., Roberts, J. D. & Van Beusichem, M. (1992). *Cancer Res.* **52**, 5065–5072. 1516063

[bb11] Farrugia, L. J. (1999). *J. Appl. Cryst.* **32**, 837–838.

[bb12] Farrugia, L. J. (2012). *J. Appl. Cryst.* **45**, 849–854.

[bb13] Giaccone, G. (2000). *Drugs*, **59**, 9–17. 10.2165/00003495-200059004-00002 10864226

[bb14] Grabner, S. & Bukovec, P. (2015). *Acta Chim. Slov.* **62**, 389–393. 10.17344/acsi.2015.1433 26085422

[bb15] Heffeter, P., Jungwirth, U., Jakupec, M., Hartinger, C., Galanski, M., Elbling, L., Micksche, M., Keppler, B. & Berger, W. (2008). *Drug Resist. Updat.* **11**, 1–16. 10.1016/j.drup.2008.02.002 18394950

[bb16] Jakupec, M. A., Galanski, M. & Keppler, B. K. (2003). *Rev. Physiol. Biochem. Pharmacol.* **146**, 1–54. 10.1007/s10254-002-0001-x 12605304

[bb17] Kasparkova, J., Marini, V., Najajreh, Y., Gibson, D. & Brabec, V. (2003*b*). *Biochemistry*, **42**, 6321–6332. 10.1021/bi0342315 12755637

[bb18] Kasparkova, J., Novakova, O., Farrell, N. & Brabec, V. (2003*a*). *Biochemistry*, **42**, 792–800. 10.1021/bi026614t 12534292

[bb19] Kelland, L. R. (2007). *Nat. Rev. Cancer*, **7**, 573–584. 10.1038/nrc2167 17625587

[bb20] Kuo, M. T., Chen, H. H., Song, I. S., Savaraj, N. & Ishikawa, T. (2007). *Cancer Metastasis Rev.* **26**, 71–83. 10.1007/s10555-007-9045-3 17318448

[bb21] Lippert, B. (1999). *Cisplatin Chemistry and Biochemistry of a Leading Anticancer Drug*. New York: Wiley-VCH.

[bb22] Macrae, C. F., Sovago, I., Cottrell, S. J., Galek, P. T. A., McCabe, P., Pidcock, E., Platings, M., Shields, G. P., Stevens, J. S., Towler, M. & Wood, P. A. (2020). *J. Appl. Cryst.* **53**, 226–235. 10.1107/S1600576719014092 PMC6998782 32047413

[bb24] Min, Y., Mao, C., Xu, D., Wang, J. & Liu, Y. (2010). *Chem. Commun.* **46**, 8424–8426. 10.1039/c0cc03108a 20936244

[bb25] Montero, E. I., Díaz, S., González-Vadillo, A. M., Pérez, J. M., Alonso, C. & Navarro-Ranninger, C. (1999). *J. Med. Chem.* **42**, 4264–4268. 10.1021/jm991015e 10514297

[bb26] Nardelli, M. (1995). *J. Appl. Cryst.* **28**, 659–659.

[bb51] Nonius (2001). *COLLECT*. Nonius BV, Delft, The Netherlands.

[bb27] Oberoi, H. S., Nukolova, N. V., Kabanov, A. V. & Bronich, T. K. (2013). *Adv. Drug Deliv. Rev.* **65**, 1667–1685. 10.1016/j.addr.2013.09.014 PMC4197009 24113520

[bb52] Otwinowski, Z. & Minor, W. (1997). *Methods in Enzymology*, Vol. 276, Macromolecular Crystallography, Part A, edited by C. W. Carter Jr & R. M. Sweet, pp. 307–326. New York: Academic Press. 10.1016/S0076-6879(97)76066-X 27754618

[bb53] Oxford Diffraction (2002). *CrysAlis*. Oxford Diffraction Ltd, Abingdon, Oxfordshire, England.

[bb54] Palatinus, L. & Chapuis, G. (2007). *J. Appl. Cryst.* **40**, 786–790.

[bb28] Rabik, C. A. & Dolan, M. E. (2007). *Cancer Treat. Rev.* **33**, 9–23. 10.1016/j.ctrv.2006.09.006 PMC1855222 17084534

[bb29] Rauko, P., Novotny, L., Dovinova, I., Hunakova, L., Szekeres, T. & Jayaram, H. N. (2001). *Eur. J. Pharm. Sci.* **12**, 387–394. 10.1016/s0928-0987(00)00180-9 11231105

[bb30] Reedijk, J. (2011). *Pure Appl. Chem.* **83**, 1709–1719.

[bb31] Rosenberg, B., Van Camp, L. & Krigas, T. (1965). *Nature*, **205**, 698–699. 10.1038/205698a0 14287410

[bb32] Sheldrick, G. M. (2010). *SADABS*. University of Gottingen, Germany.

[bb33] Sinnokrot, M. & Sherrill, C. D. (2006). *J. Phys. Chem. A*, **110**, 10656–10668. 10.1021/jp0610416 16970354

[bb34] Steward, D. J. (2007). *Crit. Rev. Oncol. Hematol.* **63**, 12–31.

[bb35] Van Beusichem, M. & Farrell, N. (1992). *Inorg. Chem.* **31**, 634–639.

[bb36] Vernhet, L., Petit, J. Y. & Lang, F. (1997). *J. Pharmacol. Exp. Ther.* **283**, 358–365. 9336344

[bb37] Vinci, D. & Chateigner, D. (2022). *Acta Cryst.* B**78**, 835–841.

[bb56] Watkin, D. J., Prout, C. K. & Pearce, L. J. (1996). *CAMERON*. Chemical Crystallography Laboratory, Oxford, England.

[bb38] Wiltshaw, E. (1979). *Plat. Met. Rev.* **23**, 90–98.

[bb39] Wong, E. & Giandomenico, C. M. (1999). *Chem. Rev.* **99**, 2451–2466. 10.1021/cr980420v 11749486

[bb40] Zhang, J., Dai, J., Lan, X., Zhao, Y., Yang, F., Zhang, H., Tang, S., Liang, G., Wang, X. & Tang, Q. (2022). *Eur. J. Med. Chem.* **233**, 114215. 10.1016/j.ejmech.2022.114215 35227978

